# Flatbed *epi* relief-contrast cellular monitoring system for stable cell culture

**DOI:** 10.1038/s41598-017-02001-x

**Published:** 2017-05-15

**Authors:** Tatsuya Osaki, Tatsuto Kageyama, Yuka Shimazu, Dina Mysnikova, Shintaro Takahashi, Shinichi Takimoto, Junji Fukuda

**Affiliations:** 1 Faculty of Engineering, Yokohama National University, Yokohama, 240-8501 Japan; 2 Optical System Development Division, R&D Group, OLYMPUS Corporation, Hachioji, 192-8507 Japan

## Abstract

Consistent cell preparation is a fundamental preliminary step for understanding complex cellular mechanisms in various cell-based research fields, including basic cell biology, cancer research, and tissue engineering. However, certain elusive factors, such as cellular de-differentiation and contamination with mycoplasma or other types of cells, have compromised the reproducibility and reliability of cell-based approaches. Here, we propose an *epi* relief-contrast cellular monitoring system (eRC-CMS) that allows images of cells in a typical culture plate to be acquired, stored, and analysed for daily cell quality control. Due to its full flatbed nature and automated system, cells placed at any location on the stage can be analysed without special attention. Using this system, changes in the size, circularity, and proliferation of endothelial cells in subculture were recorded. Analyses of images of ~9,930,000 individual cells revealed that the growth activity and cell circularity in subcultures were closely correlated with their angiogenic activity in a subsequent hydrogel assay, demonstrating that eRC-CMS is useful for assessing cell quality in advance. We further demonstrated that eRC-CMS was feasible for the imaging of neurite elongation and spheroid formation. This system may provide a robust and versatile approach for daily cell preparation to facilitate reliable and reproducible cell-based studies.

## Introduction

There is increasing concern regarding scientific research results that cannot be reproduced, particularly in the fields of basic and preclinical biological research^1^. Reproducibility is at the heart of scientific research, and misleading studies result not only in wasted valuable resources, time, and effort for follow-up studies but also in the loss of public confidence in biological and medical research^2^. Some poorly reproducible studies have been attributed to cellular de-differentiation, contamination from mycoplasma or other cell lines, misidentification of cell types, and inappropriate cell handling. There is a maximum passage number to which cells isolated from the body can be grown *in vitro* while maintaining the *in vivo* nature and characteristics of interest that are fundamental to predict *in vivo* phenomena using cultured cells. Mycoplasma contamination appears to be widespread in many laboratories, considering the fact that a broad investigation revealed that 22.4% of ~1,500 samples were contaminated with mycoplasma^3^. There is a list of more than 360 cell lines known to be cross-contaminated and misidentified^4^, and several journals have recently required or strongly recommended cell line authentication^5^. Contamination by mycoplasma and other types of cells can be inspected and eliminated with relatively little effort using fluorescent staining of mycoplasma DNA or standard molecular biology procedures, such as PCR^6^. Such an inspection should be conducted when a new cell line comes to a lab and routinely thereafter as long as the line is used for experiments. However, in reality, it is challenging to keep all cell lines authenticated for every experiment. Furthermore, there are many other potential triggers compromising studies or making non-ignorable experimental errors in the preparation of primary cells and cell lines, such as excessive pipetting of the cell suspension, non-uniform distribution of cells in a dish, and the denaturing of growth factors included in fetal bovine serum. Therefore, in addition to routine contamination inspections, an approach for the continuous monitoring of cell behaviour during subculture on a daily basis without additional intense labour may be desirable for cellular quality control in every cell culture laboratory.

Cell quality has typically been checked in culture preparations at least by counting the number of cells and observing the cellular shapes using phase-contrast microscopy because the cells exhibit specific doubling times and morphological characteristics. However, as described above, many previous publications have indicated that these manual checks of cell numbers and morphology once every few days might be insufficient for proper quality control. Continuous monitoring of cell morphology and proliferation can be performed using commercially available systems (e.g., IncuCyte, Essen BioScience, USA; BioStation, Nikon, Japan) that include an incubator box mounted on a stage of a standard inverse microscope or a standard incubator with a built-in microscope^7, 8^. However, both systems are designed for focusing on cellular events rather than for cell quality control and are unfit for the simultaneous monitoring of cells in multiple culture plates. In addition, these systems, particularly the latter, are typically very expensive. Recently, a lens-free video microscope system^9, 10^ and a compact wireless microscope system^11^ were separately reported. These systems are cost-effective and designed for the continuous monitoring and analysis of cells, but the resolutions of the systems seem to be insufficient. Microstructures such as neurites, filopodia and lamellipodia have not been visualized with these systems, which are unlike a typical phase-contrast microscope. An ideal system for quality control may be one in which (i) cells can be continuously monitored under stable culture conditions in a CO_2_ incubator without any handling of the culture plates and disturbance of the culture; (ii) no special skill or additional labour is required, with multiple culture dishes and plates placed in a CO_2_ incubator being automatically recognized, digitally labelled, and analysed in terms of cell morphology, proliferation, and other parameters; (iii) recorded image data, analysed outcomes, and alarms for cell quality are accessible online; and (iv) the system can be inserted into a typical CO_2_ incubator and can be produced inexpensively.

**Figure 1:**
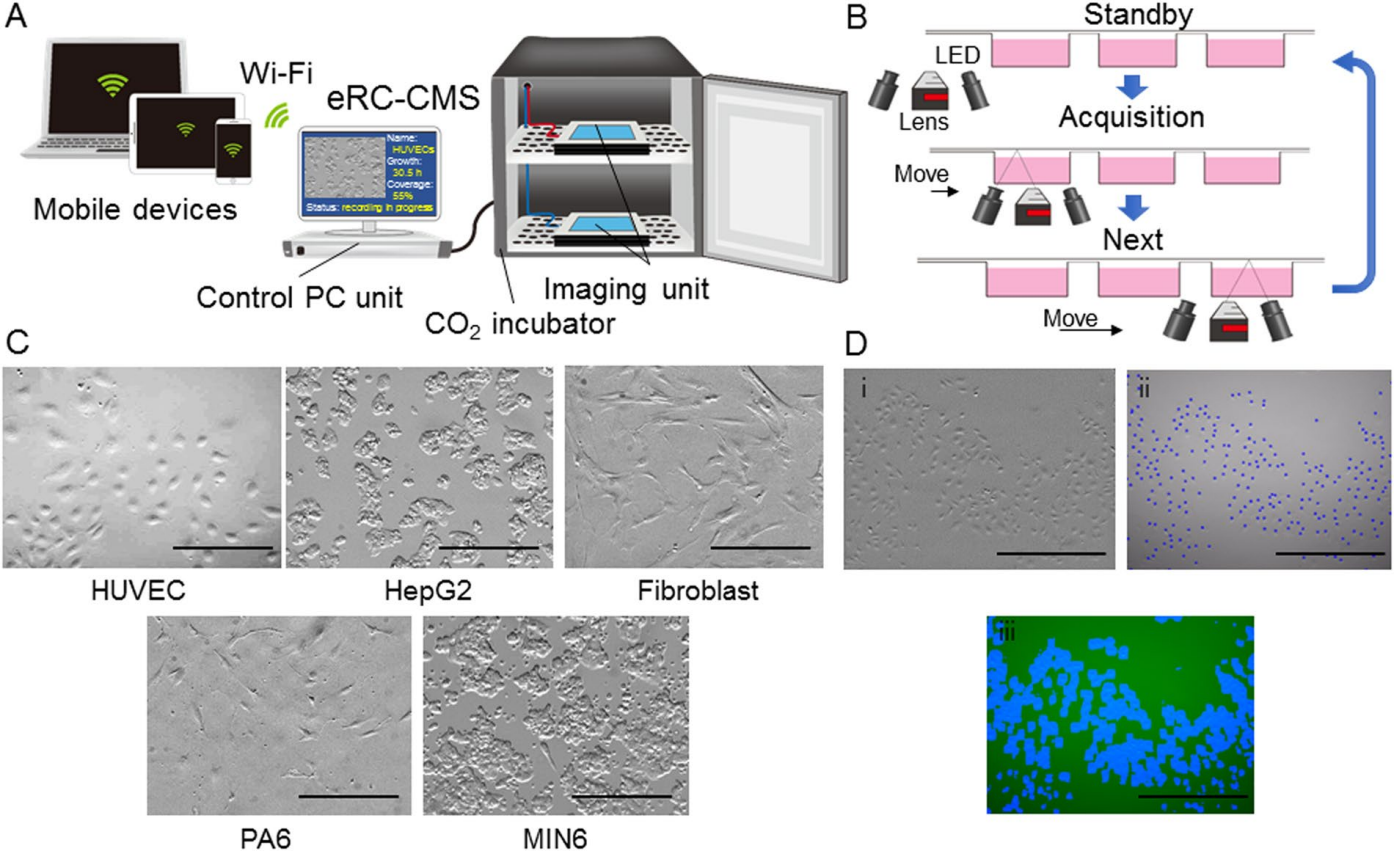
Flatbed *epi* relief contrast cellular monitoring system (eRC-CMS). (**A**) Configuration and culture scheme. eRC-CMS consisted of an imaging unit, control unit, and PC. The imaging unit was equipped with an objective lens and LEDs for *epi* relief contrast imaging. HUVEC images were automatically acquired every 60 min from 24 to 72 hours of culture in a 6-well plate. Image data were automatically processed with custom software to obtain the cell number, confluency, and cell circularity. (**B**) Examined regions. Cell images (1.2 mm × 0.8 mm) were acquired at 5 different regions in each well in a 6-well plate (a total of 30 regions/plate). (**C**) Image analysis. An acquired cell image (i) was automatically processed and labelled with blue dots for counting the cell numbers (ii) or was transformed to a binary image for the quantification of confluency (iii). Scale bars: 500 μm. (**D**) Comparisons of images of different types of cells. Scale bars: 200 μm.

In this study, we proposed an *epi* relief-contrast cellular monitoring system (eRC-CMS, Fig. 1A) for the quality control of cells grown in culture. A unique feature of eRC-CMS was that LED light sources were integrated into the same side of an objective lens and produced cell imaging with *epi*-oblique illumination, making the system flat and the imaging component fully movable in the entire area of a flat stage. We examined whether eRC-CMS could be used to monitor the behaviour of human umbilical vein endothelial cells (HUVECs) with different passage numbers and to quantify changes in morphology, size, circularity, and proliferation. These cell characteristics were linked with endothelial angiogenic activity in a subsequent hydrogel culture that is important for *in vitro* angiogenic models for the development of anti-angiogenic cancer drugs and for engineering vascularized tissues. We further examined whether eRC-CMS was capable of sufficient resolution to acquire time-lapse images of neurite elongation and three-dimensional spheroid formation.

## Results and Discussion

### System setup

The schematics of eRC-CMS are shown in Fig. 1A. The system consisted of imaging units and a control PC unit. The imaging unit was designed to be full-flat, to fit on a shelf in a CO_2_ incubator, and to work under humidified conditions. The imaging components included a CMOS camera, a lens, and LEDs that were integrated on a small board that was precisely driven in x-y directions by automated stepping motors in the imaging unit, facilitating the imaging of cells in a culture plate placed anywhere on the flat stage. In this study, the movement speeds of the imaging component board were 9.0 mm/sec (x-axis) and 12.5 mm/sec (y-axis), which was sufficient for time-lapse imaging of cells in several 6-well culture plates (see below). As shown in Fig. 1B, the *epi* relief-contrast imaging of cells was enabled by our original visualization technology using the reflection of LED light on the surface of the lid of a culture plate. The imaging unit was wired to the control PC unit to control the imaging component board in the x-y-positions and the lens in the z-position, to display a live image, and to acquire, store, and process image data. Because all operations are controllable from outside the incubator, the culture environments can remain stable. Using an image analysis algorithm (CKX-CCSW, Olympus), acquired images were automatically processed for the improvement of image contrast and the quantification of cell regions/non-cell regions, cell numbers, and locations based on luminance values. Image data were saved as grey-scale, 8-bit, 2592 × 1944-pixel files in JPEG format. Figure 1C shows representative images of five different types of cells in a typical 6-well plate. eRC-CMS was capable of capturing individual morphological characteristics; HUVECs, fibroblasts, and PA6 stromal cells presented relatively thin and spread shapes, whereas HepG2 and MIN6 cells represented more cuboidal and partially stacked features (Fig. 1C). These images show that eRC-CMS is more suitable for imaging cuboidal cells than for imaging thin and spread cells. eRC-CMS is not necessarily advantageous or disadvantageous over phase-contrast microscopy in terms of image quality (Supplemental Figure 1) but is sufficient for recognizing cells and extracting morphological characteristics for subsequent analysis. Figure 1D shows an image of HUVECs overlapped with its analysed image data. An image acquired with eRC-CMS (i) was automatically processed, and individual cells were labelled with blue dots (ii), or the image was transformed to a binary image (iii). Through these image processing steps, the number of cells, the growth rate, the confluency, and the cell size and circularity were quantified. Real-time image data and analysed results were accessible via the internet and intranet even from home.

### Changes in HUVEC proliferation during passage culture

In general, it is challenging to maintain the *in vivo* nature of primary cells after removing them from the body. Primary cells readily lose tissue-specific features and gene expression upon *in vitro* passage culture. For example, brain vascular endothelial cells lose their blood-brain barrier functions and related gene expression over time in *in vitro* culture^12, 13^, most likely because cell microenvironments in culture differ considerably from those presented *in vivo*. Thus, to understand complex biological mechanisms, it is important to previously validate that the cells to be used for the experiments at least partly maintain their original characteristics of interest. Here, as a proof of concept, we used eRC-CMS to monitor the passage culture of HUVECs and examined the correlations of their morphological and proliferative characteristics to their angiogenic activity in a hydrogel.

**Figure 2:**
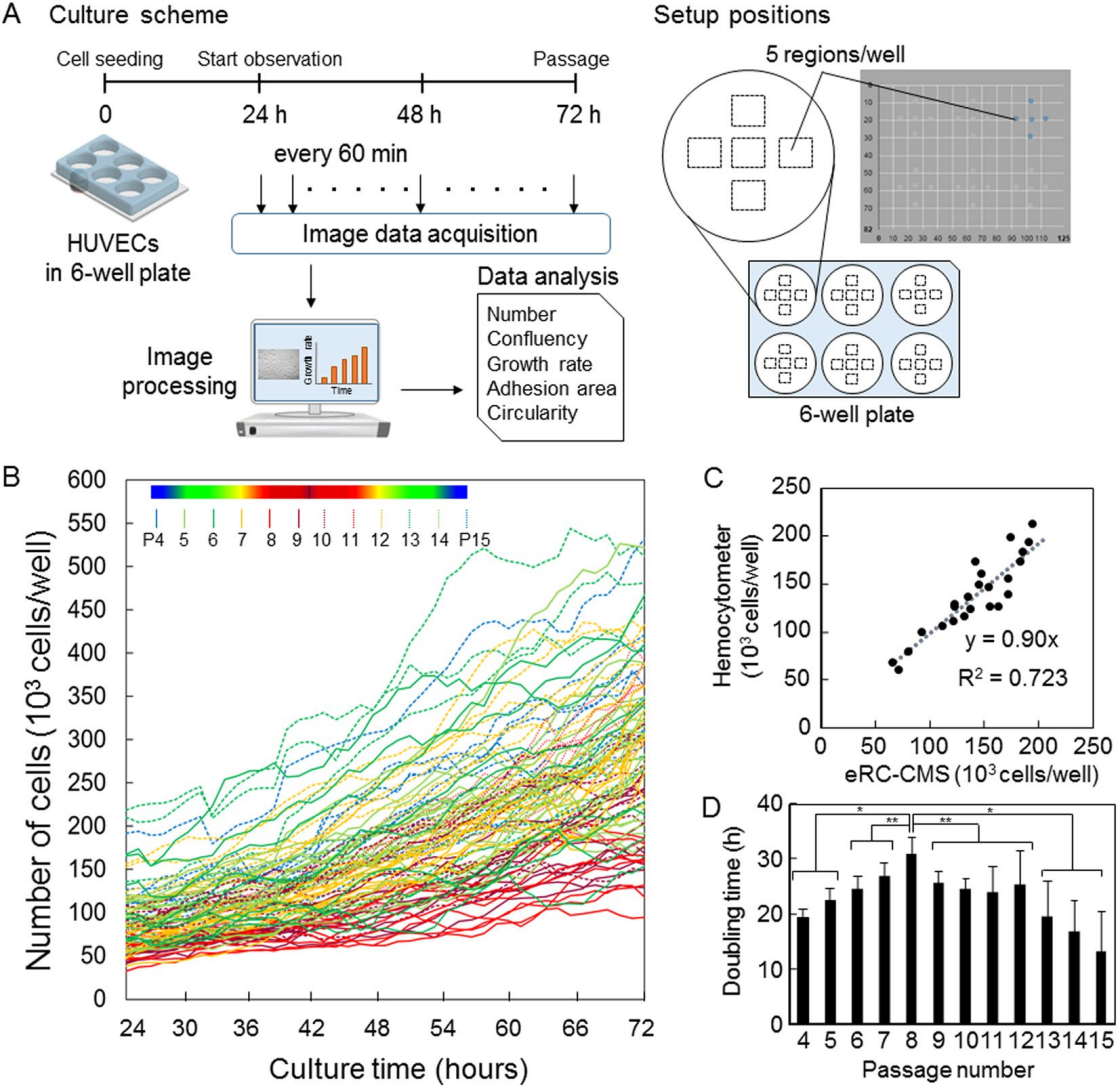
Growth activity of HUVECs at different passage numbers. (**A**) Changes in cell numbers. Using eRC-CMS, images were acquired every 60 min from 24 to 72 hours of culture at 10 different regions in two wells for each passage number. HUVECs at passages from 4 to 15 were examined. (**B**) Comparisons of the numbers of cells counted using eRC-CMS and a haemocytometer at 72 hours of culture. The digits in the graph indicate the passage number. (**C**) Doubling time. The values and error bars are averages and standard deviations calculated from the data at 24 and 72 hours in (**A**).

HUVECs are derived from the endothelium of veins from the umbilical cord and have been used as a standard model for the study of the vascular endothelial system. In the monitoring of passage culture with eRC-CMS, we obtained HUVECs at passage 4 and prepared passage 10 by repeating a standard passage culture. Cells at passages 4 and 10 were then seeded in two 6-well plates at a density of 3.0 × 10^4^ cells/well. The two plates were then placed on the eRC-CMS imaging unit in a 5% CO_2_ incubator. After 24 hours of culture, images of cells at 5 different regions per well in each of the 12 wells were taken with eRC-CMS every 60 min for the following 48 hours, as shown in Fig. 2A. HUVECs were then collected from the wells by using trypsinization and were seeded in two new 6-well plates at the same density, 3.0 × 10^4^ cells/well. These steps were repeated from passages 4 to 9 and from passages 10 to 15. Because the same passage protocol was used to prepare cells at passage 4 and cells at passage 10, we considered that the data were connected. In total, approximately 5760 images were stored, and ~9,930,000 individual cells were analysed.

The number of cells in each well was estimated from the average of 5 different regions. Changes in the cell number in 6 wells for a total of 12 passages were plotted as shown in Fig. 2B. The numbers of cells were compared with those estimated by manual counting on a haemocytometer at 72 hours of culture for each of the passage numbers in order to evaluate the image-based quantification with eRC-CMS (Fig. 2C). As shown by the slope (0.90) calculated using the least-squares method in Fig. 2C, ~10% more cells were counted by eRC-CMS than with manual counting. Although the variances between the two cell counting methods (R^2^ = 0.72) were not ignorable, we considered that counting with eRC-CMS was practical for monitoring cell proliferation behaviours. The doubling time of HUVECs was calculated from the data in Fig. 2B and is shown in Fig. 2D. The doubling time at passage 4 was ~20 h, which is comparable to previous reports in which the doubling times at passages 1 to 4 were 17 to 24 hours^14^. The doubling time increased with increasing passage number and reached a maximum at passage 8 (~31 h) but then decreased and became much shorter at passage 15 (~16 h) than at the first passage.

### Morphological characteristics of HUVECs in passage culture

**Figure 3:**
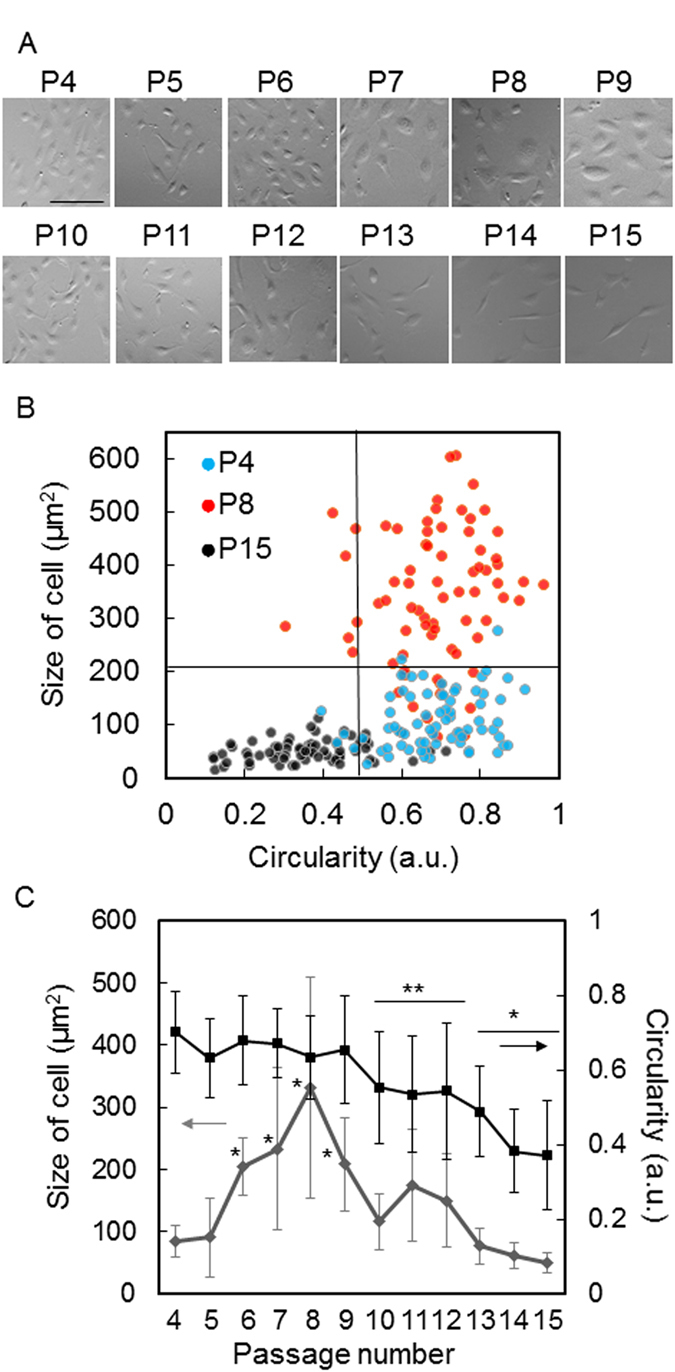
Morphological analysis of HUVECs at different passage numbers. (**A**) Morphology of HUVECs at passages 4 to 15. The images were taken with eRC-CMS at 48 hours of culture. The scale bar indicates 100 μm. (**B**) Relationship between the size and circularity of cells at passages 4, 9, and 15. At least 30 cells were analysed at each passage. (**C**) Dependence of the size of cells and circularity on passage number. The values and error bars indicate averages and standard deviations calculated from these data of at least 30 cells for each passage number (*p < 0.01, **p < 0.05, compared to passage 4).

The morphology of individual cells was monitored with eRC-CMS from passages 4 to 15, which revealed that HUVECs altered their morphological characteristics depending on the passage number. As shown in Fig. 3A, compared to passage 4, the cells exhibited spread and extended morphologies at passages 7 to 9 and spindle-shaped and fibroblast-like morphologies at passages 13 to 15. The size and the circularity of individual cells were quantified from all the eRC-CMS images. The size was expressed as the projected area of cells, and the circularity was defined as 4π × area/perimeter^2^. In Fig. 3B, the relationships between these two morphological characteristics were plotted for at least 30 randomly selected cells at passages 4, 8, and 15. There were clear associations between the passage number and both the size and the circularity. Thus, based on these morphological characteristics, the cells can be categorized into three groups: P4, round and small; P8, round and large; and P15, non-round and small. The averages and standard deviations of the size and the circularity of the cells at all of the passage numbers from 4 to 15 are shown in Fig. 3C. The size of the cells increased as the passage number increased, peaked at passage 8 (~330 μm^2^), and then decreased over the next passages. The size at passage 15 (~60 μm^2^) was much smaller than that at passage 4 (~80 μm^2^). Note that this dependence on the passage number entirely corresponds with the doubling time (Fig. 2D). In contrast, the circularity monotonically decreased as the passage number increased.

Changes in size and proliferation in *in vitro* culture have been studied in the contexts of senescence and tumourigenesis for more than four decades^15, 16^. There is substantial evidence that cellular senescence is an anti-cancer or tumour-suppressor mechanism and a contributor to ageing^17^. Cellular senescence is defined by the fact that cells taken out from the body have a limited capacity to proliferate and enter a state of cell-cycle arrest in *in vitro* culture^17, 18^. This cell-cycle arrest is accompanied by an increase in the cell size^19^. Although further studies must be performed, the decrease in proliferation and the increase in cell size in the present study may be associated with cellular senescence. In contrast, the subsequent increase in proliferation and decrease in cell size may be associated with de-differentiation and transformation.

### Angiogenic activity of HUVECs at different passage numbers

HUVECs have been used as a human model system for studying various basic aspects of endothelial functions, such as normal, abnormal and tumour-associated angiogenesis, responses to hypoxia and inflammation conditions^20–22^, and the engineering of vascularized tissues for regenerative medicine applications^23–25^. HUVECs possess the inherent ability to spontaneously form networks of capillary-like tubes on/in hydrogels such as collagen, fibrin, and Matrigel, in the presence of the appropriate growth factors, such as vascular endothelial growth factor. It is experimentally well known that this capacity will be lost upon repeated subculture, which is the primary reason for the use of HUVECs in this study.

**Figure 4:**
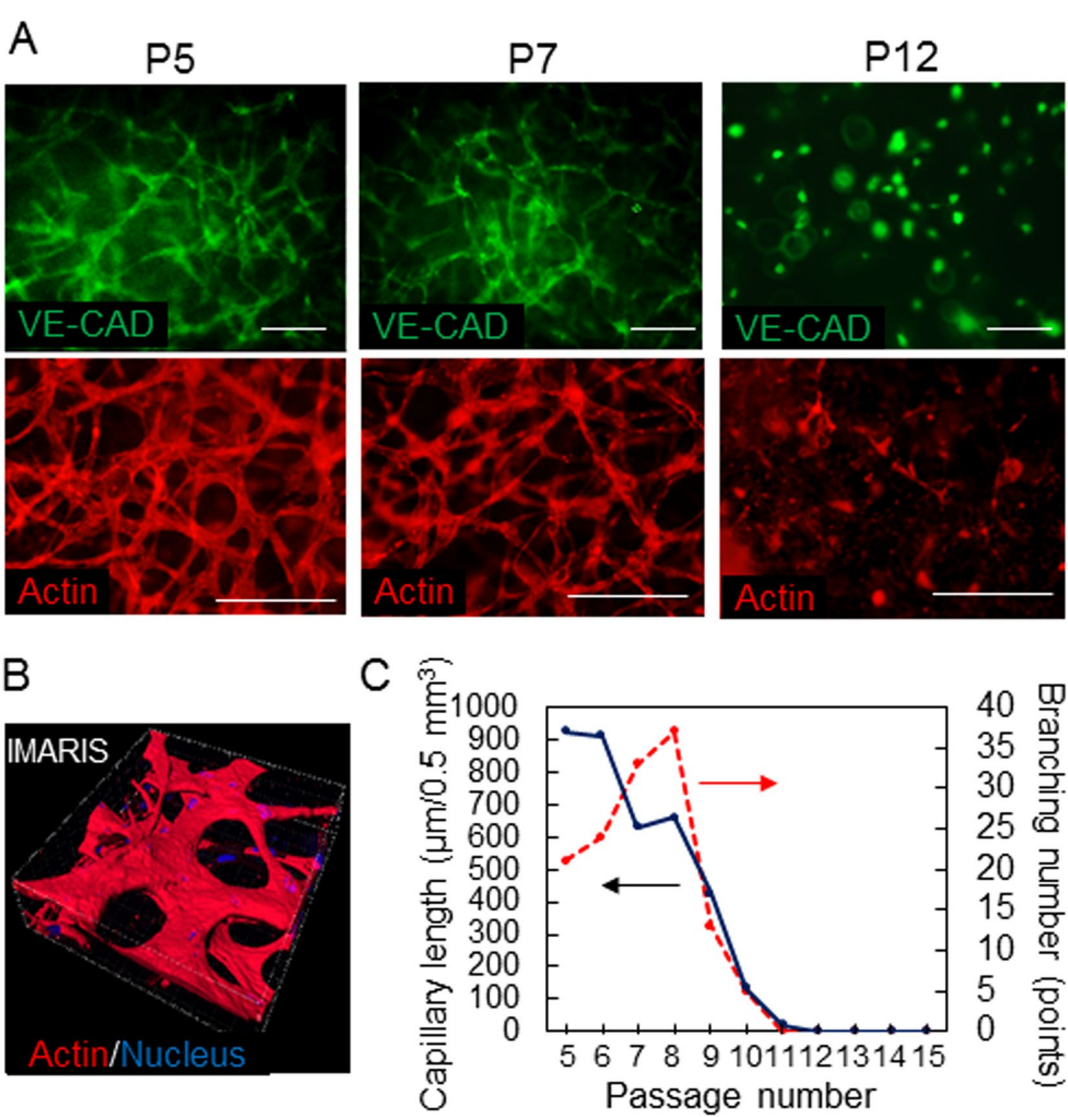
Dependence of the angiogenic activity of HUVECs on passage number. (**A**) Vascular networks. Cells at passages 5, 7, and 12 were encapsulated in a collagen gel, and VE-cadherin (Alexa Fluor 488) and actin (rhodamine-phalloidin) were stained at 48 hours of culture. The scale bar is 200 μm. (**B**) Three-dimensional configuration of vascular networks. Spatial distributions of cells at 48 hours of culture were visualized with a confocal laser-scanning microscope and were analysed using image analysis software (IMARIS). Actin (rhodamine-phalloidin) and nuclei (DAPI) were stained. (**C**) Capillary lengths and branching points were quantified using image analysis as shown in (**B**).

HUVECs at different passage numbers were encapsulated in a collagen gel, and the dependence of the passage number on the network formation activity was evaluated. Figure 4A shows representative images of HUVECs at passages 5, 7, and 12 at 48 hours after encapsulation. Vascular endothelial cadherin and F-actin were stained and visualized with a fluorescence microscope. Both staining approaches were useful for capturing features of capillary networks. The capillary formation activity was maintained well by the cells at passages 5 and 7, while the cells at passage 12 almost completely lost this activity and formed only short connections with a few neighbours. To quantitatively analyse capillary formation, three-dimensional images of capillary networks stained with rhodamine-phalloidin were acquired using a confocal laser microscope (Fig. 4B). The images were stacked to render three-dimensional structures, and the capillary length and branching number were quantified using the filament tracer module of the image analysis software (IMARIS, Bitplane, Switzerland) as described elsewhere^26^. As shown in Fig. 4C, the average capillary length of the networks gradually and almost monotonically decreased over time, and most of the cells at passages over 11 exhibited no network formation and remained as single cell dots in a collagen gel. Interestingly, the branching number increased from passages 5 to 8 and then rapidly decreased, approaching 0 for passages beyond 11. This dependence on the passage number was highly similar to those of the proliferation activity (Fig. 2D) and cell size (Fig. 4C). These results suggest that the proliferation activity and cell size could be used to judge whether HUVECs would maintain their capacity to form capillary networks in subsequent experiments.

### Time-lapse imaging of neurite elongation and spheroid formation

**Figure 5:**
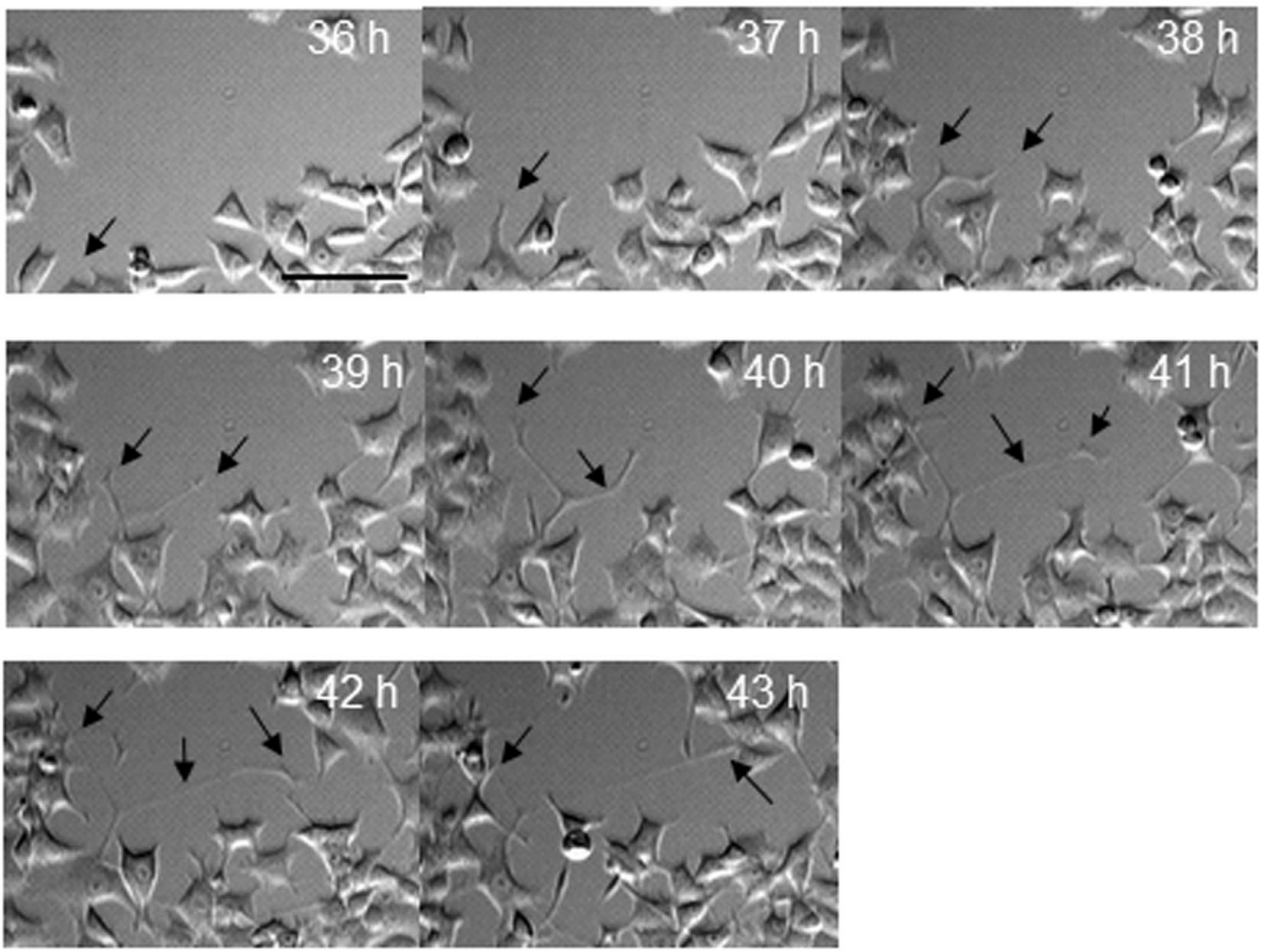
Monitoring of neurite elongation. Thin and detailed structures of elongating neurites from differentiated PC12 cells were visualized with eRC-CMS. Nerve growth factor was added to the culture medium at 24 hours of culture, and images were acquired from 36 hours to 43 hours of culture. Arrows indicate neurite- and neuronal growth cone-like structures from a single cell body. The scale bar is 50 μm.

We further investigated the feasibility of using eRC-CMS for the imaging of the thin and detailed structures observed during neurite elongation of nerve cells and of the thick and multicellular structures observed during spheroid formation. PC12 cells are derived from a pheochromocytoma of the rat adrenal medulla^27^ and have been used as a model system for neuronal differentiation and neurosecretion because these cells readily differentiate into neuron-like cells when treated with nerve growth factor^28^. PC12 cells were seeded in a typical 6-well plate, and nerve growth factor was added to the culture medium at 24 hours after seeding to initiate differentiation. As shown in Fig. 5, eRC-CMS successfully served to acquire images of neuron-like cells that were sufficiently clear to track and quantify neurite elongation. The time course of imaging at 60-min intervals revealed that neurites were induced from a cell body at 36 hours of culture (12 hours after the addition of nerve growth factor, Supplemental Figure 2). During subsequent culture, the cell body migrated a short distance, and the neurites elongated further, branched, and connected to other cells after several hours.

**Figure 6:**
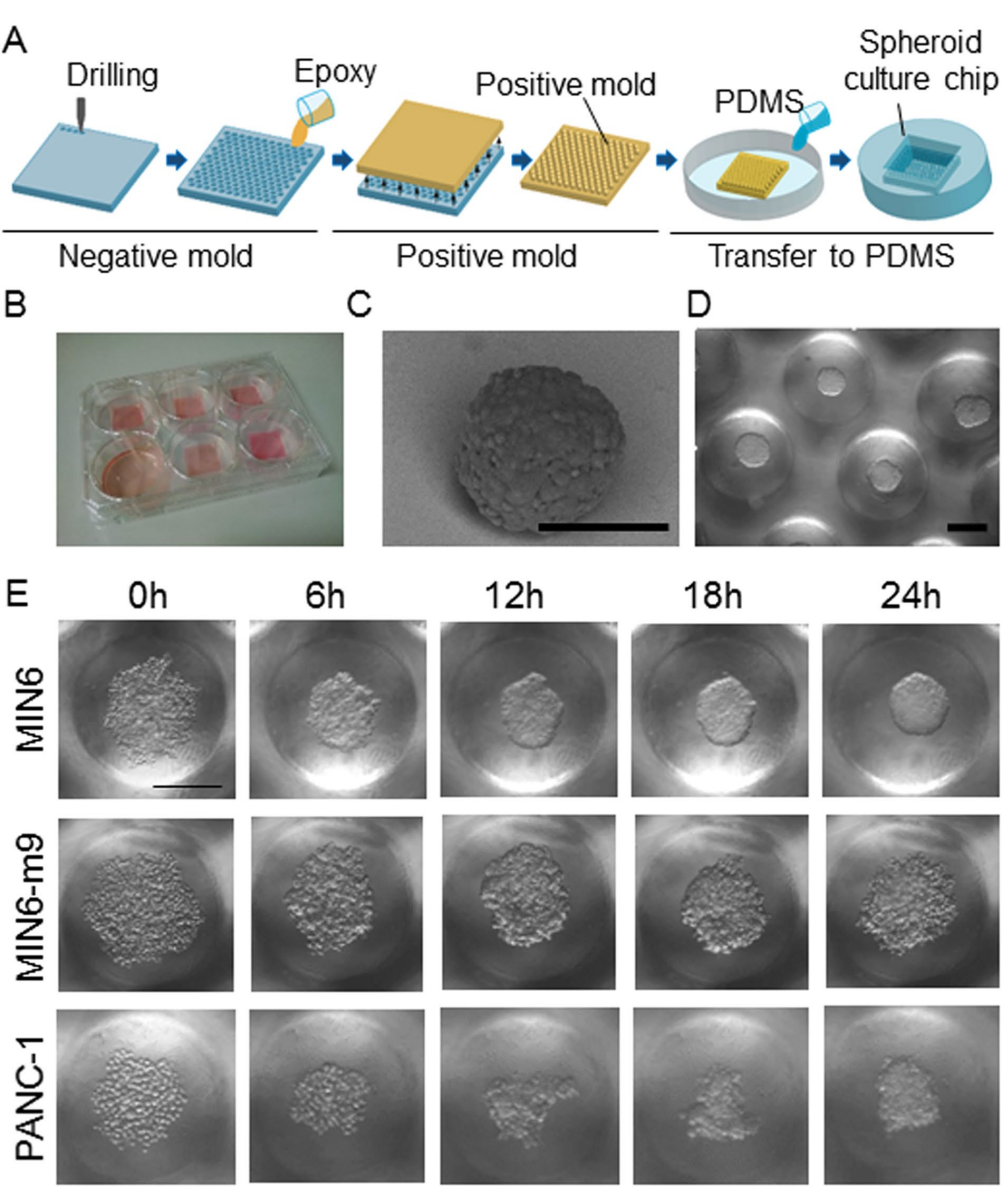
Monitoring of spheroid formation. (**A**) Schematics of the two-step moulding for fabrication of the spheroid culture chip. The chip was made of oxygen-permeable PDMS with 100 microwells (φ 500 μm). (**B**) The spheroid chips were placed in a 6-well plate. The polystyrene bottoms of the 6-well plate were removed to facilitate oxygen supply through the bottoms of the spheroid chips. (**C**) Scanning electron microscopic image of MIN6 spheroids formed on a spheroid culture chip. The scale bar is 200 μm. (**D**) Representative eRC-CMS image of MIN6 spheroids on the chip. The scale bar is 200 μm. (**E**) Time-lapse imaging of spheroid formation. MIN6, PANC-1, and MIN6-m9 cells in randomly selected microwells were continuously monitored with eRC-CMS after seeding. The scale bar is 200 μm.

To acquire time-lapse images of spheroid formation, we fabricated a spheroid array chip through two-step moulding as shown in Fig. 6A. The chip was composed of poly(dimethylsiloxane) (PDMS) with 100 round-bottom microwells of 500 μm in diameter and 600 μm in pitch (centre-to-centre of microwells). The chips were placed in a 6-well plate in which the polystyrene bottoms of the wells were removed to facilitate oxygen supply through the bottom of the PDMS (Fig. 6B). We previously reported that a similar PDMS microwell chip significantly improved the survival and functions of hepatocyte spheroids due to improved oxygen supply^29^. Here, three different pancreatic cell lines, MIN6, MIN6-m9, and PANC-1, were seeded on the chip. MIN6 is a mouse pancreatic β-cell line but contains other types of pancreatic cells^30^. MIN6-m9 is a subclone of an MIN6 cell with a high insulin secretion rate^31^. Both types of cells retain glucose-stimulated insulin secretion. PANC-1 was established from a human pancreatic adenocarcinoma and is used as a model of pancreatic cancer^32^. MIN6 cells seeded on the chip settled into the microwells and formed spheroids with uniform diameters of ~200 μm. Scanning electron microscopy revealed that MIN6 spheroids indeed formed spherical aggregates (Fig. 6C). An array of spheroids on the chip was clearly visualized with eRC-CMS (Fig. 6D). Furthermore, time-lapse imaging with eRC-CMS revealed significant differences in the dynamics of spheroid formation among the three different pancreatic cell lines (Fig. 6E, Supplemental Movies 1, 2 and 3). MIN6 cells formed loosely gathered aggregates at 6 hours of culture and compacted and smooth spherical aggregates at 24 hours of culture; in contrast, PANC-1 and MIN6-m9 cells formed loosely gathered aggregates at 24 hours of culture but were not able to maintain these structures and subsequently dissociated into single cells (Supplemental Figure 3). Considering that MIN6-m9 cells are a subclone of MIN6 cells with a high insulin secretion rate, other types of cells in the MIN6 cell line may be closely involved with smooth spheroid formation and possibly other functions.

## Conclusions

This study demonstrated that a cellular imaging system based on an *epi* relief contrast observation facilitated the monitoring of cultured cells in a typical culture plate for the daily quality control of the cells and allowed the acquisition of high-resolution images of a single cell’s neurite elongation and multicellular aggregate formation. Because light reflected at the surface of the culture plate lid was sufficient to visualize cells using our visualization technology and an algorithm for image processing, LED light sources, an objective lens, and a CMOS camera were integrated on the same board, making it possible to visualize cells in culture plates placed at any location on the flat stage. Passage cultures of HUVECs were monitored with this system from passages 4 to 15, once every hour from 24 to 72 hours of culture for each passage. The analysis of the ~9,930,000 captured individual cell images revealed that both the doubling time and the cell size peaked at passage 8, which was also the threshold for their capacity to form capillary networks in subsequent culture in a collagen gel. These findings indicate that the monitoring of cell proliferation and size in culture preparations could be a potential indicator for judging the suitability of cells for subsequent angiogenic assays.

## Materials and Methods

### Cell culture

HUVECs (passage 4, CC-2517) from Cambrex Bio Science (Walkersville, MD, USA) were maintained in endothelial basal medium (EBM-2, CC-3156) and Single Quots growth supplement (CC-4176) from Lonza (Basel, Switzerland). Human hepatic carcinoma (HepG2) cells and rat adrenal pheochromocytoma (PC12) cells were maintained in Dulbecco’s modified Eagle medium (Sigma-Aldrich, D6429) supplemented with 10% foetal bovine serum (FBS) and 1% penicillin-streptomycin. Neonatal human dermal fibroblasts and mouse bone marrow stromal cells (PA6) were maintained in minimum essential medium-α (Sigma-Aldrich, M0644) supplemented with 10% FBS and 1% penicillin-streptomycin. PC12 cells were maintained in Dulbecco’s modified Eagle medium supplemented with 10% horse serum (Sigma-Aldrich, H0146) and 1% penicillin-streptomycin and were differentiated into neuron-like cells by treatment with 50 ng/ml nerve growth factor (Sigma-Aldrich, N8133). Human pancreatic carcinoma epithelial-like cells (PANC-1) were maintained in RPMI-1640 (Sigma-Aldrich, C-4176) supplemented with 10% FBS and 1% penicillin-streptomycin. Mouse pancreatic β cell lines (Min6^33^ and Min6-m9^31^, kind gifts from Prof. J. Miyazaki at Osaka University and Prof. O. Seino at Kobe University, respectively) were maintained in Dulbecco’s modified Eagle medium supplemented with 10% FBS, 1% penicillin-streptomycin, and 6.3 mM β-mercaptoethanol (Sigma-Aldrich, M6250). All cells were cultured at 37 °C with 5% CO_2_ in a humidified incubator.

### Microfabrication of the spheroid array chip

PDMS-based spheroid array chips were fabricated using a micro-moulding technique (Fig. 6A). As a negative mould, an array of microwells (diameter: 500 µm, depth: 1000 μm) was fabricated on an olefin plate (20 × 20 mm, ZEONOR 1430 R, Zeon Co., Japan) using a computer-controlled drilling machine (MDX-540S, Roland Co., Japan). The distance between the centres of two adjacent microwells was 600 µm. To transfer the structures of the negative mould to a positive mould, a mixture of epoxy resin (epoxy:curing agent, 2:1) was poured on the negative mould and cured for 48 hours at room temperature. Then, a PDMS solution (prepolymer solution:curing agent, 10:1) was poured on the epoxy resin positive mould and cured in an oven at 80 °C for 1 hour to obtain a spheroid array chip for cell culture. The thickness of PDMS at the bottom of microwells was adjusted to approximately 1 mm. The chip was immersed in MilliQ water and sterilized with an autoclave (120 °C for 1 hour). The chip was treated with 2 ml of 4% Pluronic F-127 solution for 6 hours to make the PDMS surface repulsive to cell attachment^34^. The chips were then rinsed three times with PBS to remove excess Pluronic F-127. Three pancreatic cell lines, MIN6, MIN6-m9, and PANC-1, were seeded onto the chips at a cell density of 2.5 × 10^5^ cells/chip in 2 ml of culture medium. Cell aggregations in randomly selected regions were monitored using eRC-CMS immediately after cell seeding.

### Immunohistochemistry

Cells were fixed with 4% paraformaldehyde in phosphate-buffered saline (PBS) for 15 min and were then treated with 0.2% Triton X-100 for 5 min. Next, cells were incubated first with primary antibodies (rabbit anti-human VE-cadherin 1:200, Abcam, UK, ab33168) for 2 hours at room temperature and then with secondary antibodies (Alexa Fluor 488-conjugated goat anti-rabbit IgG (H + L) 1:500, Invitrogen, A-11008) for 2 hours at room temperature, followed by washing three times with PBS. The actin cytoskeleton and cell nuclei were stained with rhodamine-phalloidin (Cytoskeleton, Inc., USA) and 4′,6-diamidino-2-phenylindole (DAPI) for 20 min at room temperature. The capillary length and sprouting branch number of HUVECs were analysed from the reconstructed three-dimensional images by using image analysis software (IMARIS, Bitplane, Switzerland). Cells were examined using a fluorescence microscope (IX-71, Olympus, Japan) and a confocal laser-scanning microscope (LSM700, Carl Zeiss, Germany).

### Statistical analysis

The data are expressed as the mean values ± SE, and one-way ANOVA tests were performed. If significant, the data were tested with multiple comparisons tests (Tukey-Kramer post hoc tests, n = 10, 30 in Figs 2D and 3C in each passage number; **p < 0.05, *p < 0.01). The tests were performed using MATLAB software (Math Works, Worcester, MA).

## Electronic supplementary material

Supplementary information

Spheroid formation of MIN6 cells, visualized using eRC-CMS.

Spheroid formation of MIN6-m9 cells, visualized using eRC-CMS.

Spheroid formation of PANC-1 cells, visualized using eRC-CMS.
