# Supplementary material for: Flatbed epi relief-contrast cellular monitoring system for stable cell culture
Source: Sci Rep. 2017 May 15;7:1897. doi: 10.1038/s41598-017-02001-x (PMC5432522; doi:10.1038/s41598-017-02001-x)
Supplement: Supplementary file 1 — Supplementary information [file 41598_2017_2001_MOESM1_ESM.pdf]

Flatbed *epi* relief-contrast cellular monitoring system for stable cell culture

Tatsuya Osaki[1], Tatsuto Kageyama[1], Yuka Shimazu[1], Dina Mysnikova[1], Shintaro Takahashi[2], Shinichi Takimoto[2], Junji Fukuda[1*]

[1] Faculty of Engineering, Yokohama National University, Yokohama, 240-8501 Japan

[2] Optical System Development Division, R&D Group, OLYMPUS Corporation, Hachioji, 192-8507, Japan

* Corresponding author E-mail: fukuda@ynu.ac.jp (JF)

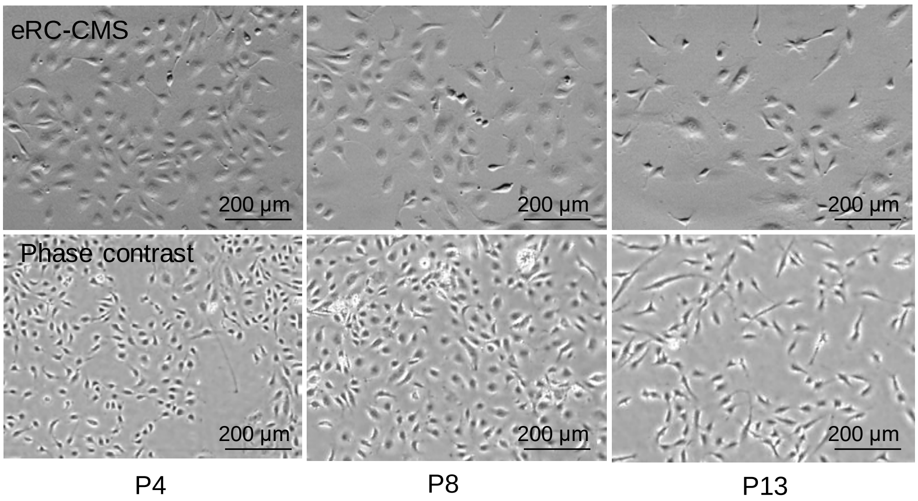

Supplemental Figure 1. Comparisons of eRC-CMS and phase-contrast microscopy. Images of HUVECs at three different passages were taken with these microscopies.

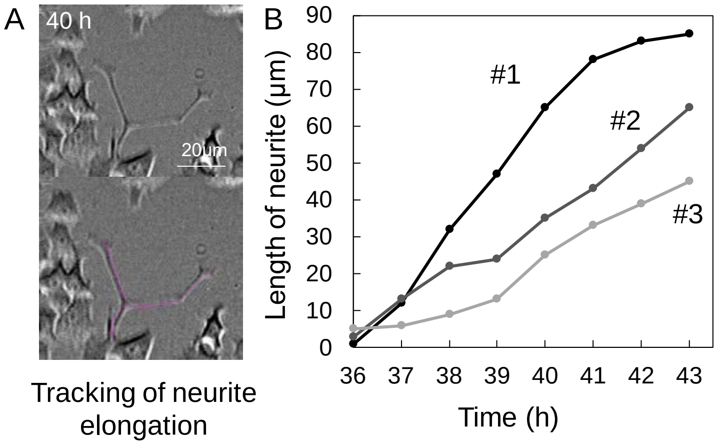

Supplemental Figure 2. Quantitative measurement of neurite length of PC12. (A) Traced line of neurite using image analysis software. (B) Time courses of neurite elongation at randomly selected three cells.

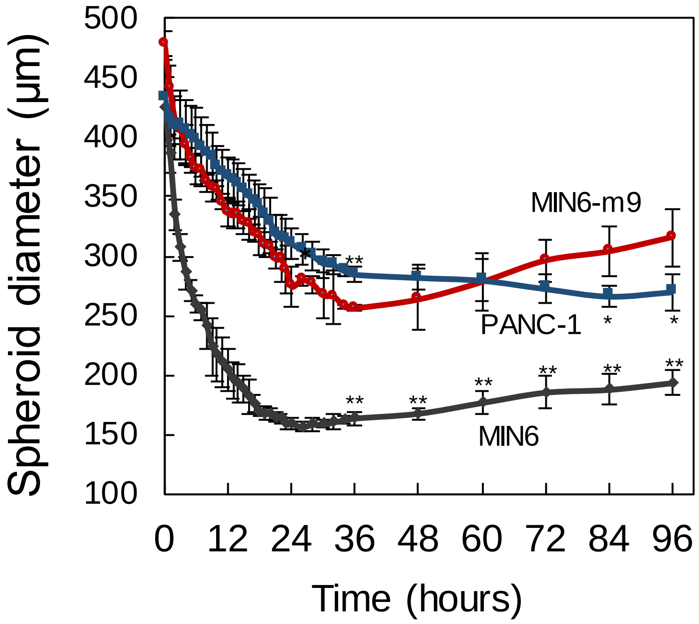

Supplemental Figure 3. Changes in spheroid diameter of three different cell lines (MIN6, MIN6-m9 and PANC-1). *p<0.05, **p<0.01, compared to MIN6-m9 on the Kruskall-Wallis one-way ANOVA test.

Caption

Supplemental movie 1. Spheroid formation of MIN6 cells, visualized using eRC-CMS.

Supplemental movie 2. Spheroid formation of MIN6-m9 cells, visualized using eRC-CMS.

Supplemental movie 3. Spheroid formation of PANC-1 cells, visualized using eRC-CMS.
